# Effect of the MOPA Laser Cleaning Parameters on the Surface Evolution of the Epoxy Coating on AH36 Steel

**DOI:** 10.3390/ma19204338

**Published:** 2026-10-15

**Authors:** Fayu Xing, Xu Du, Fachen Xu, Haoxiang Li, Ning Zhong, Yu Li, Liang Meng, Hongtao Liu

**Affiliations:** 1 Goertek College of Science and Technology Industry, Weifang University, Weifang 261061, China; 2 Yunnan Dianzhong Urban Construction Investment Development Co., Ltd., Kunming 650211, China; 3 CNOOC Energy Development Equipment Technology Co., Ltd., Tianjin 300450, China; xufch2@cnooc.com.cn; 4 Merchant Marine College, Shanghai Maritime University, Shanghai 201306, China; 5 Lumavia (Shanghai) Intelligent Technology Co., Ltd., Shanghai 201306, China; 6 Anhui Province Huaihe Vessel Inspection (Crew Management) Service Center, Bengbu 233000, China; 7 School of Machinery and Automation, Weifang University, Weifang 261061, China

**Keywords:** MOPA laser cleaning, parameter optimization, surface morphology, AH36 steel, adhesion strength

## Abstract

High-power laser cleaning has been extensively adopted to remove organic coatings, but pulse duration, pulse energy, and overlap rate must be adjusted to limit residue and thermally affected surface modification. In this work, a 1000 W MOPA pulsed fiber laser was used to strip a 75 ± 5 μm thick iron-red epoxy primer from AH36 marine steel. Single-factor tests and an L9(3^3^) orthogonal design were evaluated using image-derived paint removal rate (PRR), surface roughness (Ra), SEM morphology, and energy-dispersive X-ray spectroscopy (EDS); every processing condition was repeated on five independently prepared coupons. Increasing pulse energy from 5.0 to 12.5 mJ raised the mean PRR from 79.01% to 99.67%, although the improvement above 7.5 mJ was marginal. At 70% overlap, PRR reached 99.12%, Fe was 77.7 wt.%, C was 8.1 wt.%, and Ra reached 4.196 μm, showing that high Fe exposure alone did not define the best surface state. Descriptive range values from the replicated L9 runs gave the apparent PRR order overlap rate > power > pulse duration; since the archived worksheet retained only run means, the small differences among the ranges cannot be tested against within-run error and are not treated as statistically significant. Run 2 (300 ns, 6.00 mJ at 100 kHz, and 60% overlap) was selected from the tested combinations as a multi-index engineering compromise rather than as a predicted optimum. It produced PRR = 99.90% and Ra = 1.308 μm. Pull-off testing used three dollies per recoated condition; the mean strength of the laser-cleaned condition was 5.20 MPa and failure was predominantly cohesive within the epoxy.

## 1. Introduction

AH36 high-strength steel is extensively utilized in maritime and offshore structures, where it is typically protected by thick epoxy coatings to withstand seawater, humid salt spray, and biological fouling. During ship and offshore-structure maintenance, selective or complete removal of aged paint layers is an essential pretreatment step before recoating. Traditional paint stripping methods, including mechanical grinding, sandblasting, and chemical solvent cleaning, suffer from dust emission, abrasive consumption, operator exposure, secondary waste, and possible substrate damage [1,2]. Laser cleaning utilizes the interaction between the high-intensity pulsed photons and coating materials to achieve non-contact, spatially controllable, and solvent-free removal [1,2,3,4]. The removal of organic paint layers involves thermal ablation, polymer decomposition, plasma/shock effects, and thermo-mechanical spallation, and the relative contribution of each mechanism depends strongly on the laser fluence, pulse duration, scanning speed, and spatial overlap [5,6,7].

For high-throughput ship maintenance, kilowatt-class laser systems are attractive, but high average power can also intensify thermal accumulation, surface oxidation, and local melting when the process window is not properly selected [4,8,9,10]. Therefore, the cleaning endpoint should be evaluated by both visible coating removal and surface integrity, not by area-based removal rate alone. The MOPA (Master Oscillator Power Amplifier) pulsed fiber laser provides additional process flexibility because pulse duration and repetition frequency can be programmed over a wide range. This makes it possible to tune peak power, single-pulse energy, and cumulative thermal dosage independently, which is especially useful for thick organic coatings whose removal requires a balance between surface ablation and interfacial spallation [11,12].

Several studies have addressed parts of this problem. Ma et al. [8] varied laser power, repetition rate/pulse duration, and spot overlap during Nd:YAG cleaning of oxide layers on Q345 steel and evaluated oxygen content, roughness, microhardness, and electrochemical behavior. Their selected condition (280 W, 10 kHz, and 70% overlap) minimized the surface oxygen content and improved corrosion resistance, whereas excessive heat input produced ablation pits and new oxides and reduced corrosion performance. However, the contaminant was a corrosion oxide layer rather than a thick pigmented polymer coating, and the 280 W regime did not resolve the scaling of heat accumulation in kilowatt-class MOPA cleaning. Wang et al. [5] combined a simplified three-dimensional thermal model with moving-pulse experiments and found that an overlap ratio above 50% generated a more uniform heated zone without obvious paint residue, while stripping depth increased approximately linearly with incident energy density. Nevertheless, that study focused on thermal distribution and removal depth and did not jointly assess residual carbon/oxygen, substrate roughness, or recoating performance. More recently, Sonoiki et al. [9] used femtosecond laser-induced breakdown spectroscopy (LIBS) and compared the spectra with those generated during high-power continuous-wave removal of marine coatings; layer-specific spectral signatures allowed different coating layers to be identified and their ablation times to be estimated. Although this work demonstrated the potential of spectroscopic endpoint monitoring, it did not establish a closed-loop criterion for nanosecond MOPA processing or correlate the optical endpoint with PRR, SEM/EDS, Ra, and recoating adhesion. Thus, a multi-index process window for kilowatt-class MOPA removal of thick marine epoxy coatings remains insufficiently defined.

However, laser cleaning ultimately aims to secure durable recoating performance. Liu et al. [13] reviewed micro/nanocarrier-based self-healing anticorrosive coatings and emphasized that coating durability depends on barrier continuity, inhibitor release, and defect repair rather than appearance alone. Wang et al. and Ma et al. [14,15] incorporated Fe_3_O_4_-MBT nanoparticles or magnetically guided microcapsules into epoxy systems, showing improved barrier/self-healing behavior in NaCl media, but their studies optimized protective coating formulations instead of the cleaned steel surface produced before recoating. Pan et al. [16] used deep-learning super-resolution to recognize the micromorphology of epoxy coatings in deep-sea environments, while Wang et al. [17] showed that seawater freeze-thaw cycles increased porosity and accelerated chloride ingress in epoxy coating on EH36 ship steel. These studies underline the need to link laser-cleaning parameters with residue/oxide control and recoating-relevant surface integrity, which remains insufficiently addressed for kilowatt-class MOPA cleaning of marine epoxy primers.

The industrial objective of laser paint stripping is not merely to remove the visible coating, but to obtain a chemically and morphologically acceptable steel surface while limiting excessive oxidation and thermal effects. Since high PRR values may still be accompanied by oxidation, carbonized residue, redeposition, or surface roughening, the cleaning endpoint should be evaluated using a combined criterion of PRR, Ra, SEM, EDS, and recoating pull-off performance [6,8,9,10].

In this work, we investigated the process window and surface evolution of a 1000 W MOPA pulsed fiber laser for stripping a 75 μm thick iron-red epoxy primer from AH36 marine high-strength steel. The specific objectives are: (i) to identify a practical processing window and a multi-index selection criterion through single-factor and orthogonal experimental designs; (ii) to characterize the surface morphology, chemical composition, and micro-roughness evolution of the AH36 substrate under different laser parameter combinations; and (iii) to interpret the likely contributions of photothermal ablation and thermo-mechanical spallation during thick-coating removal. The outcomes are intended to provide practical engineering data for the high-power MOPA laser maintenance of marine steel structures.

## 2. Experimental

### 2.1. Materials

The substrate material used in this study was AH36 marine high-strength steel supplied by Kunshan Development Zone Deliao Metal (Kunshan, China), which was machined into rectangular specimens with dimensions of 50 × 50 × 5 mm. AH36 steel is a widely used structural steel grade specified for ship hull applications by classification societies, with excellent weldability and adequate toughness in marine environments. The nominal chemical composition is listed in Table 1.

Prior to coating application, all specimens underwent a rigorous surface pretreatment procedure to replicate standard shipyard practice. The steel surfaces were abrasive blasted using brown fused alumina grit to achieve a surface cleanliness level of Sa 2.5 (ISO 8501-1 [18]) and a surface profile (Rz) of 40–70 μm (ISO 8503-2 [19]). Subsequently, the blasted specimens were immersed in an acetone bath and ultrasonically cleaned for 15 min, followed by drying at 60 °C for 30 min.

The iron-red epoxy coating, as a representative of the standard heavy-duty anticorrosive coatings used in marine engineering, was uniformly applied by spray coating. The main chemical constituents are listed in Table 2. The primer was cured at ambient temperature (23 ± 2 °C, RH ≤ 65%) for 168 h (7 days). The dry film thickness was measured at 25 randomized locations per specimen using a calibrated digital coating thickness gauge (Elcometer 456, Elcometer Limited, Manchester, UK), and the average thickness was controlled to 75 ± 5 μm, providing a consistent sample set for the laser cleaning experiments.

### 2.2. MOPA Pulsed Fiber Laser Cleaning System

**Figure 1:**
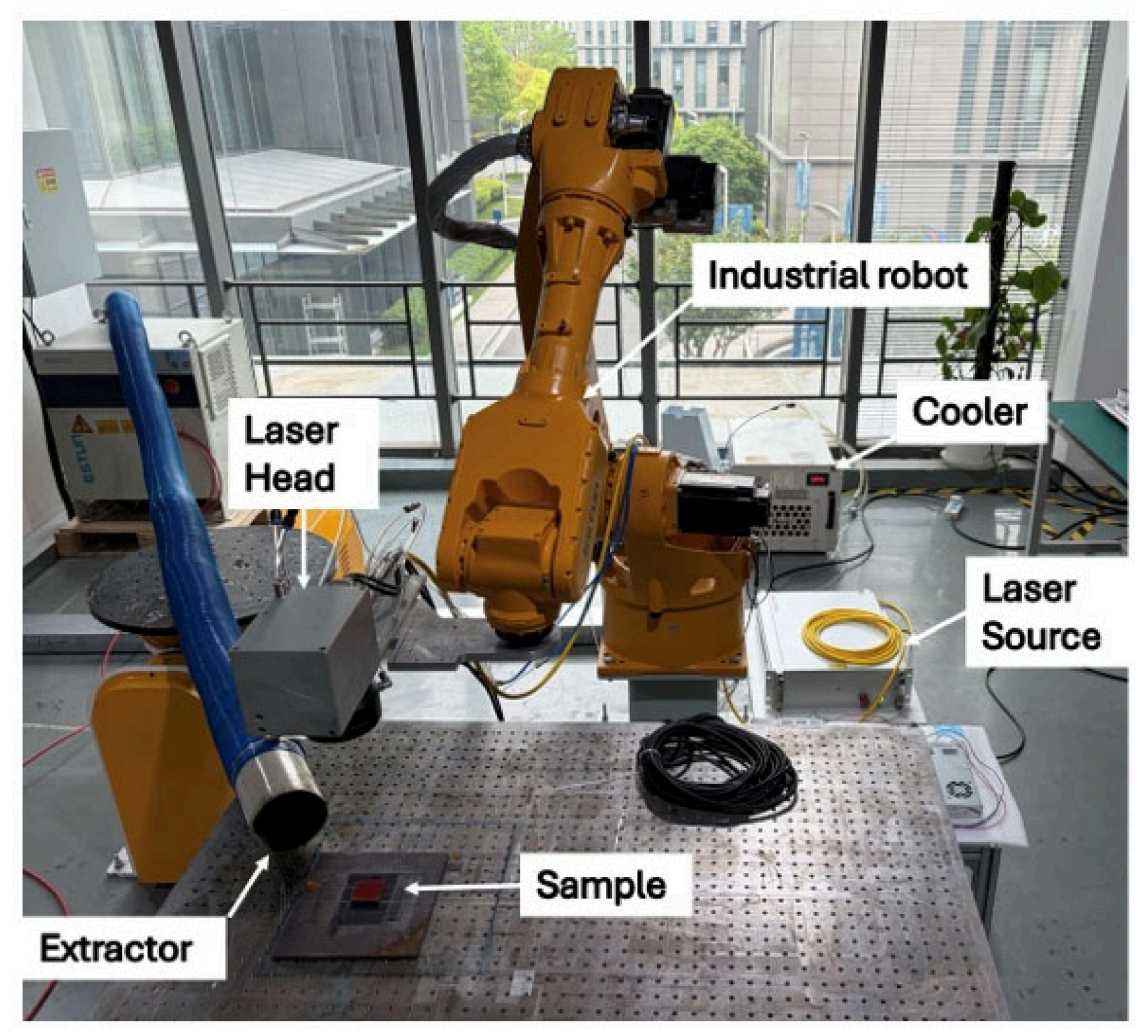
Experimental setup of the 1000 W MOPA pulsed fiber laser cleaning platform.

The paint stripping experiments were conducted using a MOPA pulsed fiber laser system. Table 3 summarizes the key optical and operational parameters of the laser system. As a fundamental advantage of the MOPA architecture, the pulse duration (100–500 ns) and repetition frequency (66–1000 kHz) can be independently programmed, enabling flexible and precise control over both the peak power and the temporal energy distribution delivered to the coating surface. Figure 1 shows the actual cleaning platform, including the laser source, fiber delivery, galvanometer cleaning head, sample stage, and control cabinet used during the experiments.

The laser beam was delivered through a QBH fiber output coupler and directed onto the sample surface via a 2D galvanometer scanning head. An F-theta scanning lens with a focal length of 160 mm was used. The lens-to-surface working distance was 160 mm, and the beam was incident normal to the specimen surface (90° to the specimen plane). The effective focused spot diameter (D) was estimated from the hatch-spacing/overlap relationship. With L_y_ = 0.125 mm and η_y_ = 50%, η_y_ = 1 − L_y_/D gives D = L_y_/(1 − η_y_) = 0.125/0.5 = 0.25 mm (250 μm). The laser fluence (E_d_), defined using the circular spot area, is:

(1) $$E_{d}=\frac{4E}{\pi D^{2}}$$

where E is the single-pulse energy, and D is the focused spot diameter. The reported values were calculated after converting D to centimeters. For example, at E = 5.0 mJ and D = 250 μm = 0.025 cm, E_d_ = 4(0.005 J)/[π(0.025 cm)^2^] = 10.2 J/cm^2^. The same calculation gives 25.5 J/cm^2^ at 12.5 mJ; thus, the experimental range was approximately 10.2–25.5 J/cm^2^.

The 250 μm value was treated as a nominal effective diameter for all parameter combinations because the focal geometry was unchanged. The spot was not independently profiled at every pulse duration, repetition frequency, and power setting; consequently, possible changes caused by beam quality or thermal-lensing effects are not included in the fluence uncertainty and constitute a limitation of the present calculation.

Laser interaction with the epoxy generated gaseous decomposition products and ejected particulate or condensable material, which were removed by the local extractor shown in Figure 1. Such a transient plume can attenuate or scatter the incident beam and can contribute to redeposition. Plume emission and attenuation were not measured in this study, so their effects are included implicitly in the observed process response rather than separated quantitatively. The normal-incidence laboratory geometry also does not represent the oblique incidence that may occur on complex industrial components.

**Figure 2:**
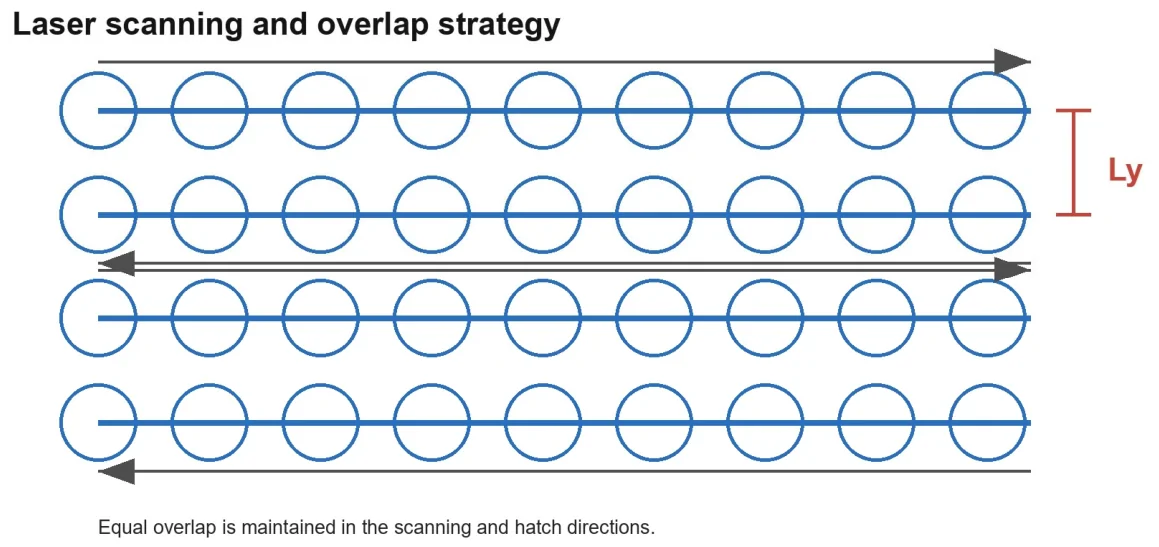
Schematic diagram of the laser scanning strategy and overlap-rate control. The gray arrows indicate the scanning directions, the blue horizontal lines represent the scanning tracks, and the blue circles schematically represent successive laser spot positions. L_y_ denotes the hatch spacing, namely the center-to-center distance between adjacent scanning lines in the slow-axis direction. The schematic is not drawn to scale; the circle spacing does not represent the actual experimental overlap.

The overlap rates in the fast-axis and slow-axis directions are defined as:

(2) $$\eta_{x}=1-\frac{V_{x}}{D\cdot f_{\mathrm{rep}}},$$

(3) $$\eta_{y}=1-\frac{L_{y}}{D},$$

where V_x_ is the scan speed in the fast-axis direction (mm/s), f_rep_ is the pulse repetition frequency (Hz), and L_y_ is the hatch spacing (mm). In the present experiments, the longitudinal and lateral overlap rates were kept equal (η_x_ = η_y_ = η) by adjusting the galvanometer scan speed V_x_ proportionally to the hatch spacing L_y_, as shown in Figure 2. This strategy maintained isotropic energy coverage across the substrate surface. The single-pulse energy E was adjusted by modulating the laser output power percentage at a fixed repetition frequency.

### 2.3. Experimental Design

The experiments were organized in two stages. Firstly, single-factor tests were used to identify feasible ranges of pulse duration, single-pulse energy/power, and overlap rate. Secondly, these screened ranges were used to construct an L9(3^3^) orthogonal design for joint evaluation. Paint removal rate was used as the primary removal-efficiency index, while the surface roughness, morphology, and EDS composition were used to judge whether a nominally cleaned surface was accompanied by residue, oxidation, redeposition, roughening, or pronounced thermal effects.

The orthogonal factors and levels were selected from the practical single-factor window rather than from the full equipment range. The 60% overlap level was introduced as an intermediate level between the effective-removal region observed at 50% and the roughening-prone condition observed at 70%, so the L9 array focuses on parameter combinations with practical cleaning potential.

Each single-factor and L9 parameter combination was applied to five independently prepared coated coupons. The PRR was calculated from one thresholded digital image of each processed coupon using ImageJ (version 1.54g), and the values reported in the figures and tables are arithmetic means of the five coupons. The archived image-analysis worksheet retained the run means but not the five individual PRR values; consequently, between-coupon standard deviations cannot be reconstructed or plotted retrospectively. Surface roughness was measured from two to three valid parallel traces on a representative coupon for each condition; the error bars reported for surface roughness represent within-surface trace variation and not between-coupon process repeatability. Since the image thresholding can classify carbonized or oxygen-bearing residue as exposed steel, PRR values such as 99.90% should not be interpreted as exact proof of complete coating removal. The close orthogonal range values are therefore treated descriptively rather than as statistically distinct effects.

### 2.4. Characterization Methods

The cleaned surfaces were characterized using scanning electron microscopy (SEM, TM3030, Hitachi High-Technologies Corporation, Tokyo, Japan) and energy-dispersive X-ray spectroscopy (EDS, Swift 3000, Oxford Instruments, Oxford, UK). SEM was used to examine residual coating, pits, re-solidified features, and redeposited material. EDS was acquired as full-field elemental area maps, not point analyses, at an accelerating voltage of 20 kV, a map resolution of 128 × 128 pixels, and a field width of approximately 1.547 mm (processing-time setting 4). One representative mapped field was quantified for each reported condition. Fe and C were used as comparative indicators of substrate exposure and organic residue, respectively; O was treated only as an oxygen signal because EDS does not identify chemical state or distinguish newly formed steel oxide from residual Fe_2_O_3_ pigment. Elemental compositions are reported to one decimal place together with the quantification software’s 1σ uncertainty where this value was exported. These σ values describe counting and peak-fitting uncertainty for the mapped field; they are not coupon-to-coupon standard deviations and do not quantify spatial heterogeneity.

Recoating performance was evaluated by the pull-off method in accordance with ISO 4624:2016 [20]. After surface preparation, the sandblasted reference, mechanically ground reference, and recommended laser-cleaned AH36 specimens were recoated with the same iron-red epoxy primer and cured under identical conditions. Three independently positioned circular steel dollies (n = 3) with a diameter of 20 mm were tested for each surface condition. The dollies were bonded to the coating using a two-component structural epoxy adhesive. After the adhesive had fully cured, the coating around each dolly was isolated by annular cutting, and a tensile load normal to the surface was applied at a controlled rate until failure. Pull-off strength was determined from the maximum tensile load divided by the dolly area, and the three values were averaged for each condition. The exposed fracture surfaces were then examined visually to distinguish adhesive failure at the steel–coating interface from cohesive failure within the coating.

## 3. Results and Discussion

### 3.1. Effect of Individual Processing Parameters on Paint Removal Efficacy

To establish a practical processing window and understand the individual contribution of each MOPA laser parameter, three single-factor experiment groups (A, B, C; Table 4) were conducted. PRR and Ra were used as complementary evaluation metrics. The factor settings and the corresponding PRR and surface roughness results for the L9(3^3^) orthogonal experiments are summarized in Table 5.

#### 3.1.1. Influence of Pulse Duration

**Figure 3:**
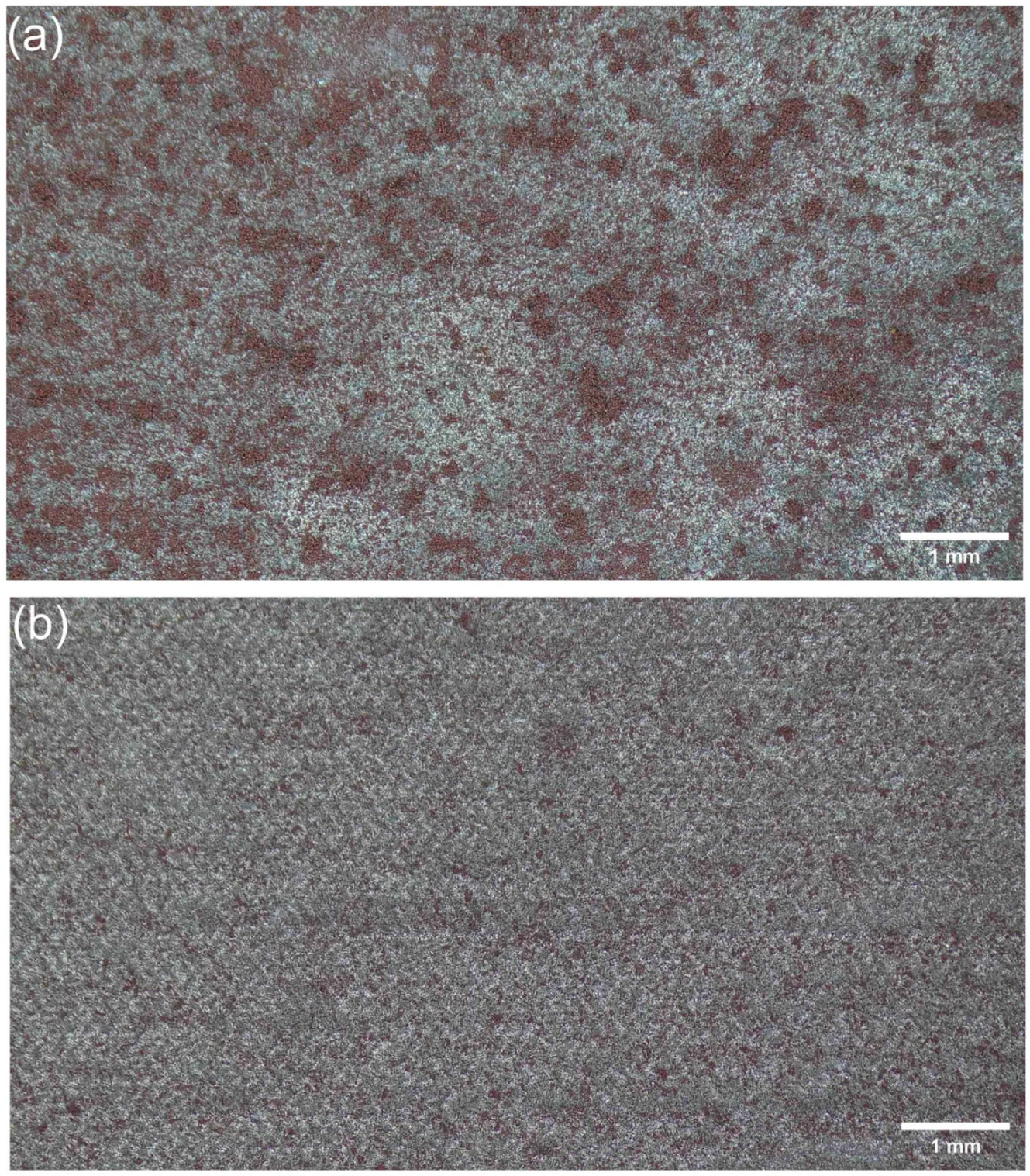

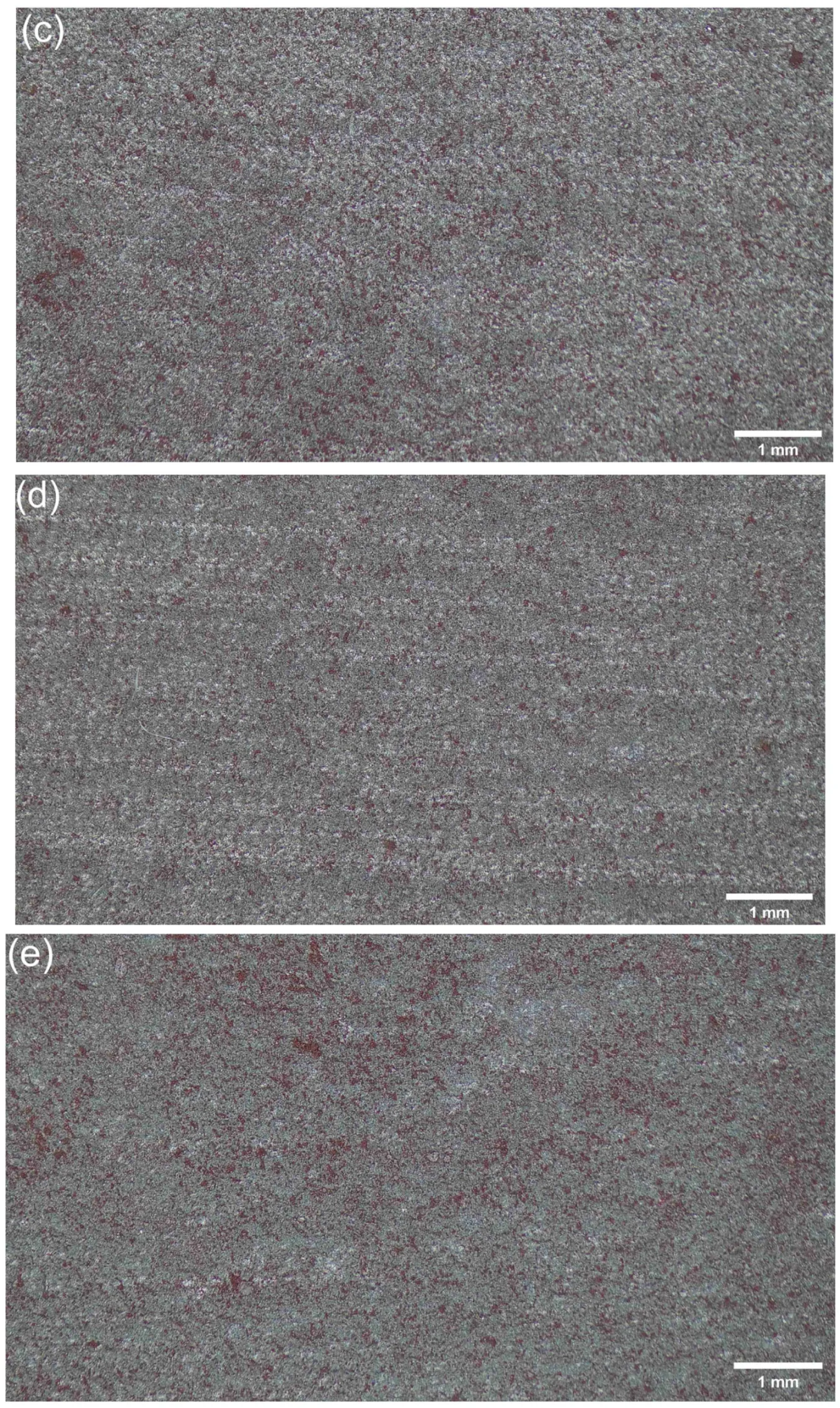
Effect of pulse duration on macroscopic cleaning morphology (E = 6.25 mJ, η = 50%): (**a**) 100 ns; (**b**) 200 ns; (**c**) 300 ns; (**d**) 400 ns; and (**e**) 500 ns.

A key advantage of the MOPA architecture is the ability to independently tune pulse duration from 100 to 500 ns while keeping the single-pulse energy constant at E = 6.25 mJ (Ed ≈ 12.7 J/cm^2^) by adjusting the repetition frequency and power proportionally. Figure 3 presents the cleaning results as a function of τ, with η = 50% held constant.

At the shortest pulse duration of τ = 100 ns, the corresponding peak power per pulse is extremely high (~62.5 kW for E = 6.25 mJ), and the interaction is dominated by high-intensity photo-ablation and plasma formation. The thermal diffusion length in the epoxy during the pulse is Lth = 0.1 μm, resulting in highly localized material removal per pulse. While ablation is energetic, the very short pulse duration limits heat diffusion into the coating bulk, resulting in moderate PRR (∼55–65%) due to insufficient cumulative penetration of the 75 μm film, and a comparatively high Ra value of 1.816 μm due to irregular local ablation and residual coating texture and local ablation craters. The macro-photograph at this condition (Figure 3a) shows conspicuous residual iron-red regions interspersed with randomly distributed ablation spots, consistent with stochastic plasma ejection rather than uniform spallation [21,22].

As τ increases to 200–300 ns, the peak power decreases while the duration of heat input increases, allowing the removal mode to shift from shallow surface ablation toward mixed ablation–spallation. The EDS data do not follow a strictly monotonic chemical trend because each value represents one mapped field. The full set of area-map results (wt.% ± software-reported 1σ) was: 100 ns, Fe 33.9 ± 0.4, C 39.0 ± 0.7, and O 27.1 ± 0.5; 200 ns, Fe 47.3 ± 0.7, C 31.5 ± 0.9, and O 21.2 ± 0.6; 300 ns, Fe 44.7 ± 0.5, C 34.7 ± 0.7, and O 20.5 ± 0.5; 400 ns, Fe 51.0 ± 0.6, C 29.8 ± 0.7, and O 19.2 ± 0.4; and 500 ns, Fe 34.3 ± 0.7, C 40.1 ± 1.0, and O 25.6 ± 0.8. These values describe analytical uncertainty within each mapped field, not repeat-coupon variability. The intermediate pulse widths are consistent with, but do not by themselves prove, greater substrate exposure [22,23].

At τ = 400 ns, PRR reached the highest mean value in the pulse-duration series (97.49%). Together with the mapped composition listed above and the macroscopic appearance, this result is consistent with effective coating disruption; it does not establish a unique removal mechanism [21,23].

At τ = 500 ns, mean PRR decreased to 93.19%. The higher mapped C signal at this condition is consistent with residual or carbonized coating. Its O signal may arise from residual Fe_2_O_3_ pigment, thermally formed steel oxide, oxygen-bearing redeposited products, or a combination of these sources. Therefore, the pulse-width window should be judged by PRR, morphology, roughness, and elemental signals together.

#### 3.1.2. Influence of Single-Pulse Energy

**Figure 4:**
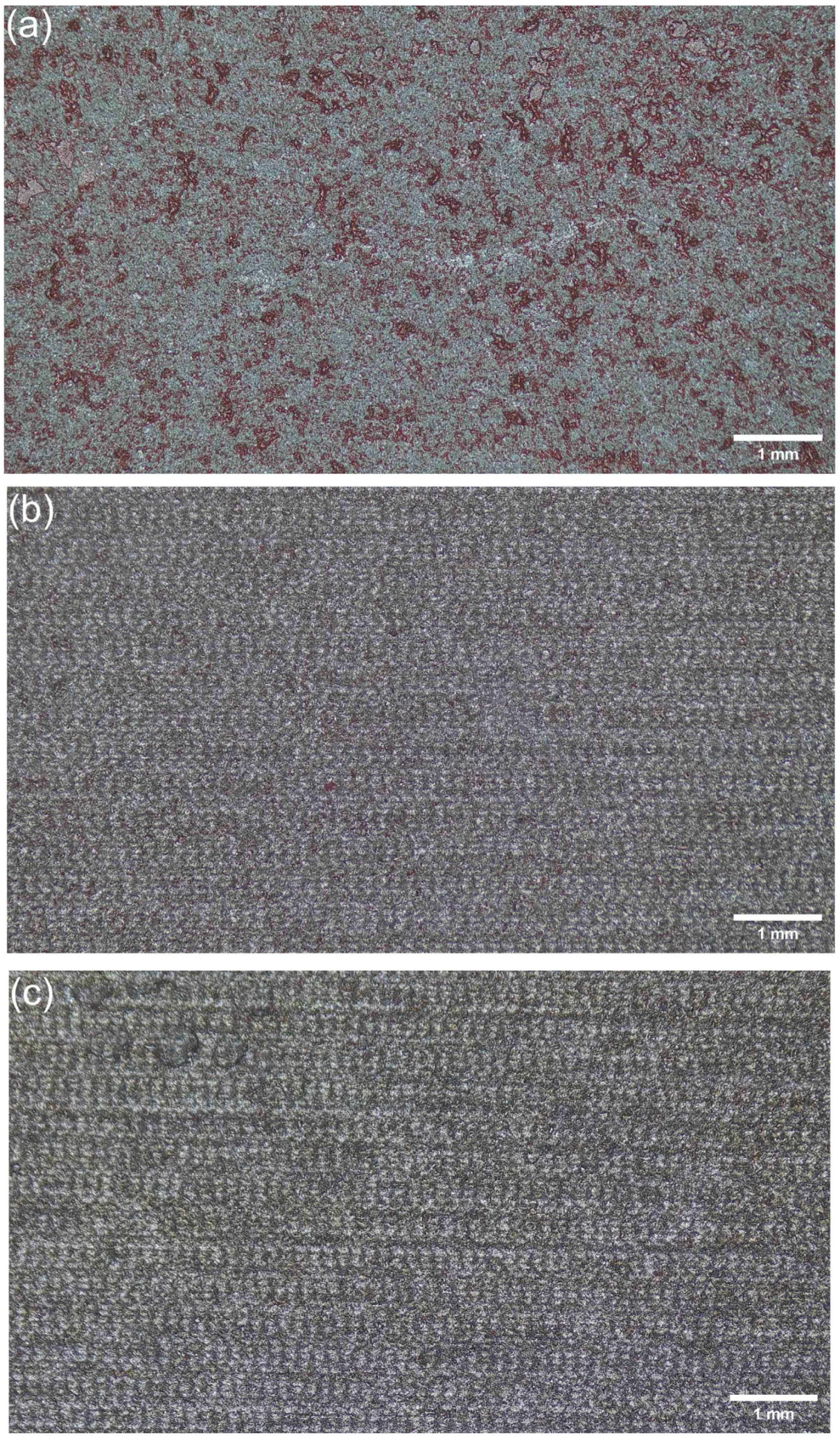

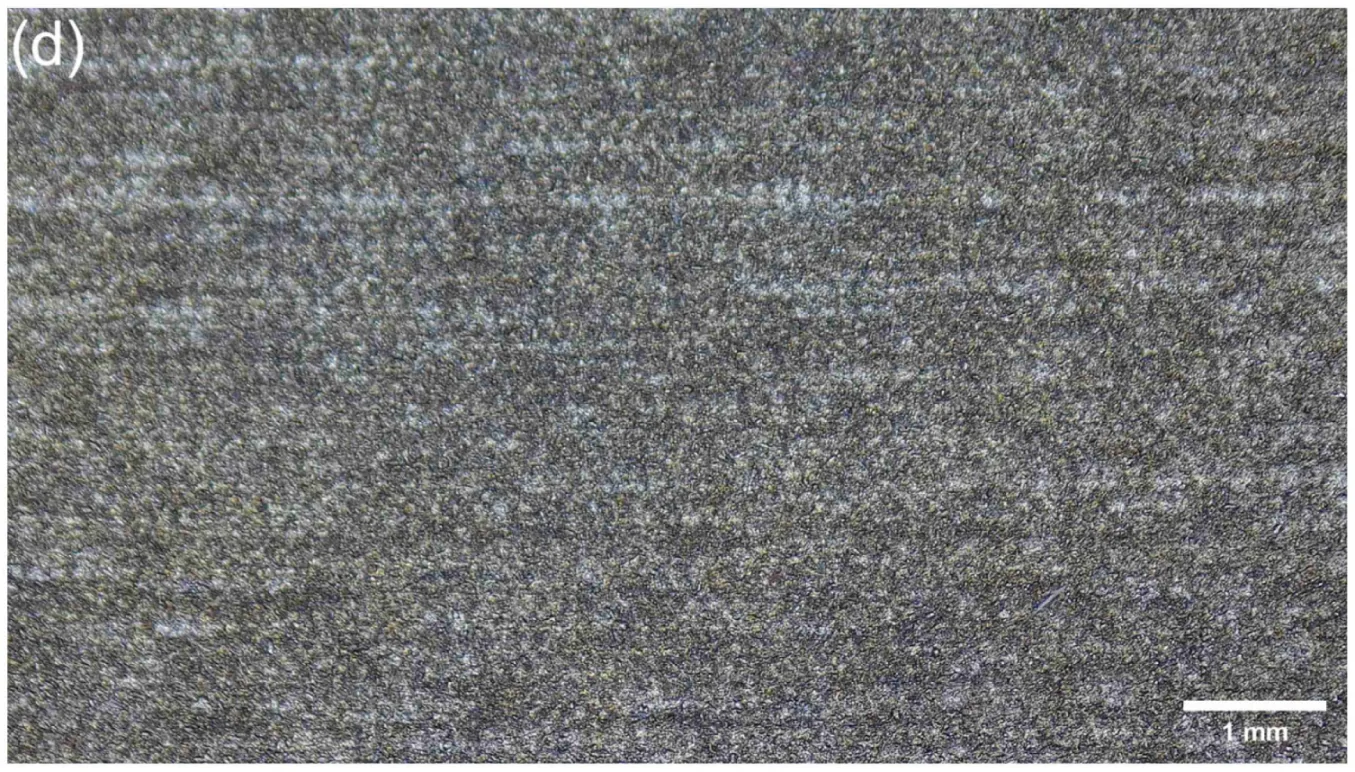
Macroscopic surface morphologies at different pulse energies (tau = 400 ns, η = 50%): (**a**) 5.0 mJ; (**b**) 7.5 mJ; (**c**) 10.0 mJ; and (**d**) 12.5 mJ.

Single-pulse energy is a key parameter in the ablation behavior because it determines whether the delivered fluence exceeds the coating-removal threshold. For the iron-red epoxy primer employed in this study, the threshold fluence was estimated from the lowest-energy condition showing measurable material removal, yielding E_d_ ≈ 8–9 J/cm^2^ (corresponding to E ≈ 3.9–4.4 mJ at the 250 μm spot diameter). Figure 4 illustrates the macroscopic surface morphologies and corresponding PRR values of AH36 specimens treated at pulse energies from 5.0 to 12.5 mJ (power percentages of 40–100% at τ = 400 ns, f = 80 kHz; E_d_ ≈ 10.2–25.5 J/cm^2^), with η = 50% held constant. The four energy levels span a range from marginally above the estimated coating-removal threshold to a high-fluence regime where additional energy mainly increases thermal loading rather than cleaning efficiency.

At the lowest energy (E = 5.0 mJ, E_d_ ≈ 10.2 J/cm^2^, power = 40%), the delivered fluence was insufficient for complete removal of the 75 μm epoxy primer in a single pass. Mean PRR was 79.01%, and the selected EDS area contained C = 45.7 wt.%, O = 29.3 wt.%, and Fe = 25.0 wt.%, consistent with a mixed surface of residual or carbonized coating and partially exposed steel. The archived B-1 export did not contain the software-calculated σ values, so analytical uncertainty cannot be reconstructed for this field.

As E increased to 7.5 mJ (E_d_ ≈ 15.3 J/cm^2^, power = 60%), mean PRR rose sharply to 98.92%. The selected EDS area contained Fe = 60.1 wt.%, C = 19.3 wt.%, and O = 20.6 wt.%, consistent with greater substrate exposure and reduced organic residue. The archived B-2 export likewise did not contain software-calculated σ values. This condition marks the observed transition from incomplete removal to high area-based removal within the tested series.

At E = 10.0–12.5 mJ (E_d_ ≈ 20.4–25.5 J/cm^2^, power = 80–100%), mean PRR increased only slightly from 99.36% to 99.67%. For 10.0 mJ, the mapped composition was Fe 62.4 ± 0.7 wt.%, C 19.1 ± 0.8 wt.%, and O 18.4 ± 0.5 wt.%; for 12.5 mJ, it was Fe 66.6 ± 0.7 wt.%, C 14.6 ± 0.8 wt.%, and O 18.9 ± 0.4 wt.% (software-reported 1σ). Because the PRR improvement was marginal, the additional fluence mainly increased energy input rather than producing a demonstrable gain in removal efficiency [23,24].

At the highest energy (E = 12.5 mJ, power = 100%), PRR was highest in this series, while the selected EDS area contained O = 18.9 wt.%. This O signal alone cannot establish laser-induced oxidation because the primer contains Fe_2_O_3_. The condition was therefore assessed together with SEM morphology and roughness for evidence of thermally affected or redeposited material.

#### 3.1.3. Effect of Overlap Rate on Paint Removal Quality and Surface Elemental Composition

**Figure 5:**
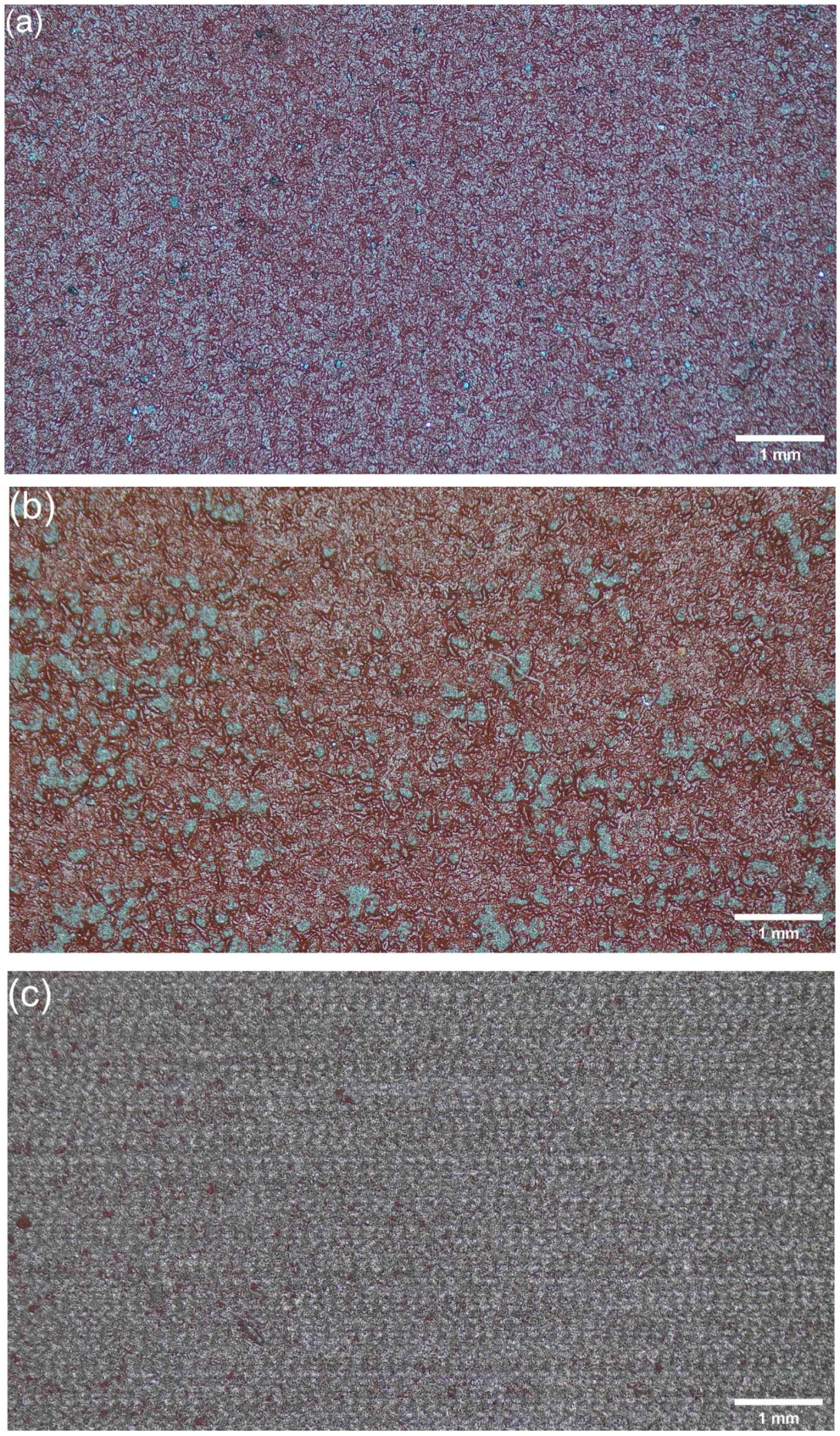

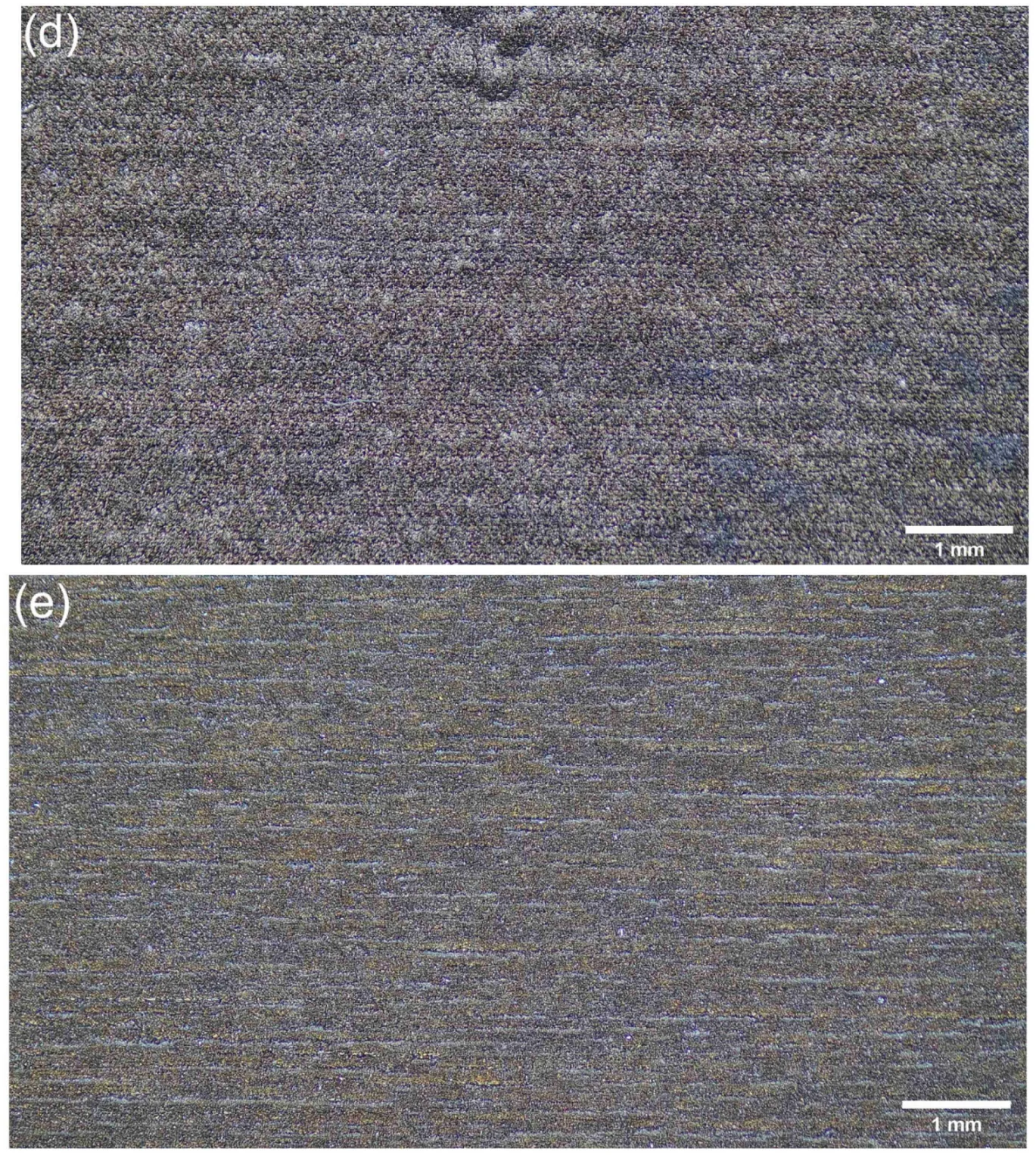
Macroscopic surface morphologies at different overlap rates: (**a**) 10%; (**b**) 30%; (**c**) 50%; (**d**) 70%; and (**e**) 90%.

The overlap rate is a key parameter governing the spatial uniformity of laser energy deposition and the cumulative energy input per unit area. In this study, the overlap rates in the scanning and hatch directions were kept identical (ηx = ηy = η) by synchronously adjusting the scan speed and hatch spacing. Figure 5 presents the macroscopic appearance and paint removal rate (PRR) obtained at different overlap rates, while the corresponding EDS results are summarized in Table 6.

At an overlap rate of 10%, pronounced unprocessed gaps remained between adjacent scanning tracks. Large areas of the iron-red primer were still visible, and PRR was only 33.05%. The selected EDS area contained Fe = 4.0 wt.%, C = 56.3 wt.%, and O = 39.7 wt.%, consistent with extensive residual coating.

When the overlap rate increased to 30%, PRR remained low at 30.42%. The selected EDS area contained Fe = 3.9 wt.%, C = 55.1 wt.%, and O = 40.9 wt.%. Thus, 30% overlap was still insufficient to establish a continuous removal front, and most of the iron-red epoxy primer remained on the surface.

At an overlap rate of 50%, PRR increased sharply to 97.72%; the selected EDS area contained Fe = 54.7 wt.% and C = 1.8 wt.%. The O signal remained high at 43.4 wt.%. Because the primer contains 25–35 wt.% Fe_2_O_3_ pigment, and because laser heating may also oxidize exposed steel, the EDS result cannot distinguish residual pigment from newly formed oxide. XPS or XRD would be required to identify chemical states or oxide phases.

At an overlap rate of 70%, PRR reached 99.12%, the highest value in this series. The selected EDS area contained Fe = 77.7 wt.%, C = 8.1 wt.%, and O = 14.2 wt.%. The high Fe signal is consistent with substantial substrate exposure; however, C was higher than at 50% overlap, and Ra increased sharply to 4.196 μm. Thus, carbonaceous residue, redeposition, or pronounced roughening may remain despite the high PRR. This condition should not be regarded as optimal on the basis of PRR or Fe alone [5,25].

When the overlap rate was further increased to 90%, PRR decreased to 92.21%. The selected EDS area contained Fe = 65.5 wt.%, C = 2.2 wt.%, and O = 32.3 wt.%. These results indicate that increasing the overlap rate to 90% did not further improve coating removal. Heat accumulation and redeposition may contribute to this behavior, but the EDS O signal alone cannot establish laser-induced oxidation without phase-sensitive analysis [4,5,25].

Overall, overlap rate exerted a dual effect on laser paint removal. Low overlap rates of 10–30% left most of the coating unremoved because adjacent tracks did not provide sufficient spatial continuity. Increasing overlap to 50–70% promoted coating detachment and increased PRR and Fe exposure, whereas excessive overlap was associated with roughening and redeposited or oxygen-bearing residual material. Overlap should therefore be evaluated using PRR, Fe/C/O signals, roughness, and SEM morphology together; EDS O content should not be equated directly with newly formed oxide.

### 3.2. Range Analysis and Apparent Interaction Analysis of the Orthogonal Experiment

#### 3.2.1. Orthogonal Experimental Results

Based on the single-factor results, pulse duration, laser power, and overlap rate were selected as the three principal factors for the orthogonal experiment. An L9(3^3^) orthogonal array was adopted to evaluate the combined influence of these parameters on paint removal, and each of the nine combinations was processed on five independently prepared coupons. Pulse duration, power, and overlap rate are denoted as factors A, B, and C, respectively. The mean paint removal rate of the five coupons was used as the main response index for quantifying the macroscopic stripping efficiency under different parameter combinations.

**Figure 6:**
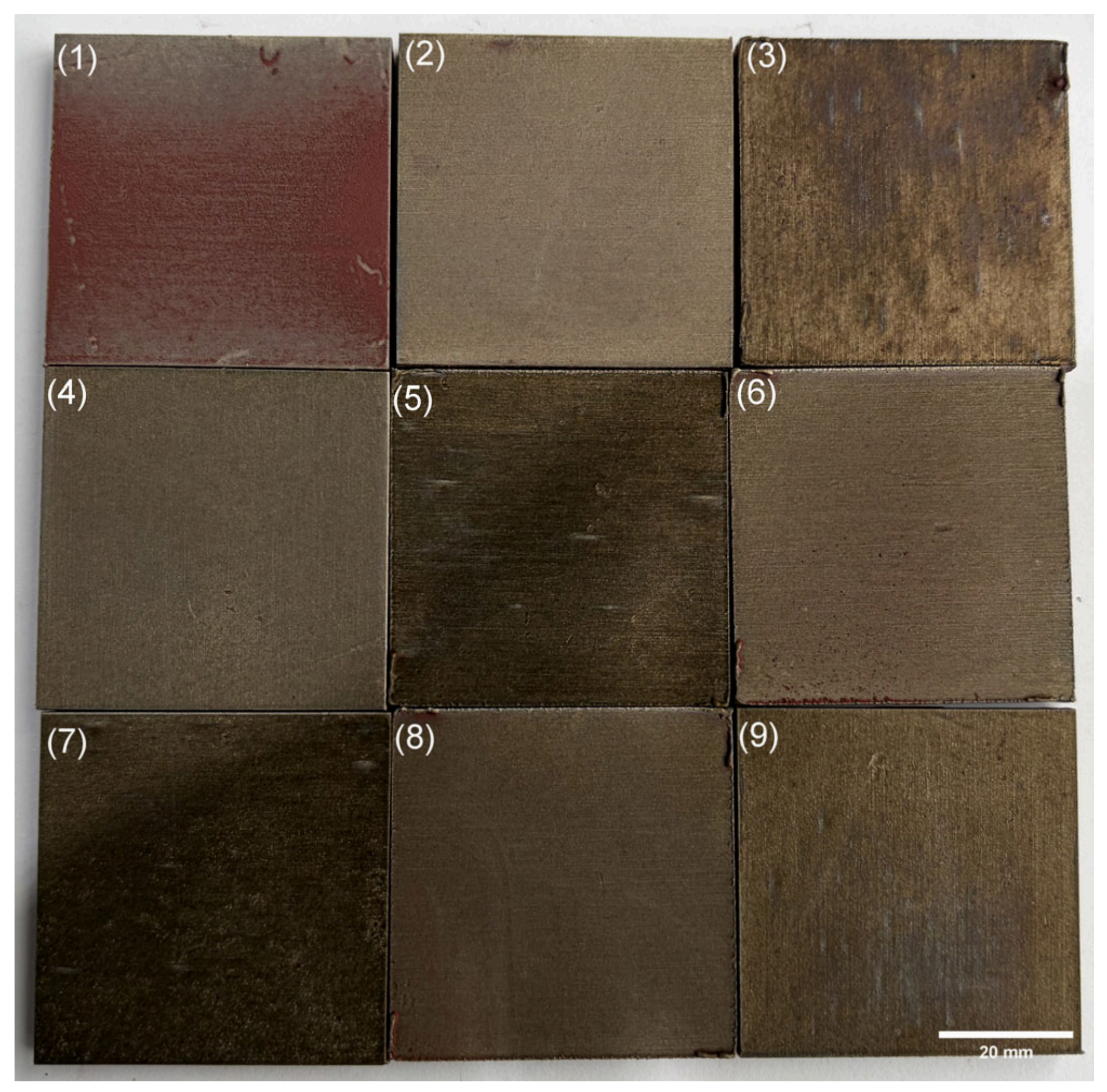
Representative laser-cleaned AH36 specimens from the L9 orthogonal experiment, showing the macroscopic surface states used for PRR evaluation. Panels (**1**–**9**) correspond to experimental runs 1–9, respectively, in Table 5, which lists the pulse duration, laser power, and overlap rate for each run. Scale bar: 20 mm; all panels are shown at the same scale.

The orthogonal run means show clear differences among the nine tested parameter combinations. Run 1 (A1B1C1) produced a mean PRR of 80.86%, indicating insufficient input for complete removal under the combined lower factor levels. Runs 2, 4, 8, and 9 produced high mean PRR values, consistent with crossing an effective removal threshold. High PRR values do not, however, imply identical surface quality because roughness, redeposition, oxygen-bearing residue, and other thermally affected features can differ. PRR must therefore be interpreted together with Ra, EDS, and SEM. Representative appearances are provided in Figure 6.

#### 3.2.2. Range Analysis

To quantify the relative influence of each parameter on PRR, range analysis was performed on the orthogonal experimental results. The mean PRR at each factor level and the corresponding range value are listed in Table 7.

As shown in Table 7, the descriptive range values give the apparent PRR order C (overlap rate) > B (power) > A (pulse duration): 6.77%, 6.30%, and 6.02%, respectively. Although each L9 combination was tested on five independently prepared coupons, the individual PRR values were not retained in the archived worksheet. Consequently, within-run experimental error cannot be estimated from the available run means, and the small differences among these ranges cannot be tested for statistical significance. The ordering therefore describes this data set only and should not be interpreted as proof that the three effects differ. Physically, overlap controls spatial coverage and repeated exposure, but confirmation of its relative importance requires replicated experiments or a design that provides an error term.

The descriptive range for power is slightly lower than that for overlap, but power remains important because it affects whether pulse energy exceeds the coating-removal threshold. Increasing power from 50% to 60% raised average PRR from 93.16% to 99.46%, whereas increasing it to 70% did not produce a further increase. Once sufficient energy is supplied, additional power provides limited removal benefit and may intensify thermally induced surface modification [23,24].

The descriptive range for pulse duration is 6.02%. Pulse duration changes peak power and the duration and depth of heat transfer. Shorter pulses favor high instantaneous intensity and surface ablation, whereas longer pulses can promote heat transfer toward the coating-substrate interface and thermo-mechanical spallation. Excessively long pulses may also increase heat input to the steel and the risk of local micro-melting or oxygen-bearing surface products. Pulse-duration selection therefore balances removal efficiency, interfacial stress generation, and substrate protection [4,21].

Although the PRR-only range analysis suggests A3B2C2 (500 ns, 60% power, and 60% overlap) and the Ra-only analysis suggests A2B2C2 (400 ns, 60% power, and 60% overlap), neither predicted combination was used as the final recommendation. A1B2C2 (300 ns, 60% power, and 60% overlap) is identical to the already tested Run 2, which produced PRR = 99.90% and Ra = 1.308 μm. It was selected from the nine measured combinations as a practical compromise based on high PRR, acceptable Ra, SEM appearance, comparative Fe/C/O signals, and lower cumulative thermal input. It is not an extrapolated optimum, and its robustness has not been confirmed by independent validation runs [26].

#### 3.2.3. Apparent Interaction Analysis

Each L9 combination was repeated on five independent coupons. In principle, these repeats can provide an experimental-error estimate; however, only the run-mean PRR values were retained in the archived analysis worksheet. The present range analysis therefore cannot separate main effects from the within-run error or interaction terms. Accordingly, the following discussion is qualitative; no statistical significance is assigned to the apparent interactions, and the small differences among factor ranges are not interpreted as a definitive ranking.

Firstly, pulse duration and power show a threshold-type coupling. At 300 ns, increasing the power from 50% to 60% increases PRR sharply from 80.86% to 99.90%, indicating that the lower power level cannot provide sufficient pulse energy for coating removal at this shorter pulse duration. Once the power reaches 60%, PRR is nearly saturated, and increasing power to 70% does not produce further improvement. By contrast, at pulse durations of 400 and 500 ns, PRR remains high across different power levels, suggesting that longer pulses enhance heat transfer into the coating and strengthen interfacial thermal stress, thereby reducing the sensitivity of PRR to power variation.

Secondly, power and overlap rate exhibit an energy-compensation relationship. At lower power, greater overlap can increase cumulative energy input and improve PRR. For thick epoxy primer, single-pulse energy and spatial repetition jointly determine whether the effective removal threshold is reached. This compensation is not unlimited: when power and overlap are both high, removal may remain efficient while the risks of redeposition, roughening, and other thermally induced surface modification increase. Thus, the data suggest a practical window below which residue remains and above which surface modification becomes more likely.

Thirdly, pulse duration and overlap rate are coupled through thermal accumulation. Longer pulses extend the thermal action time, while higher overlap increases repeated irradiation of the same region. At moderate levels, this favors continuous interfacial stress and coating spallation. At excessive levels, heat can accumulate in the substrate and increase the likelihood of roughening, re-solidification, oxygen-bearing surface products, and redeposition [4,5,25]. Therefore, long pulse duration should not be combined with excessive overlap when damage-controlled cleaning is required.

In summary, the three parameters do not act independently. Power primarily affects whether the single-pulse input exceeds the removal threshold, overlap controls spatial coverage and cumulative exposure, and pulse duration regulates the temporal distribution and penetration of heat. Their coordinated selection is essential for high PRR while limiting roughening, redeposition, oxygen-bearing residue, and excessive thermal modification.

### 3.3. Surface Roughness Evolution

**Figure 7:**
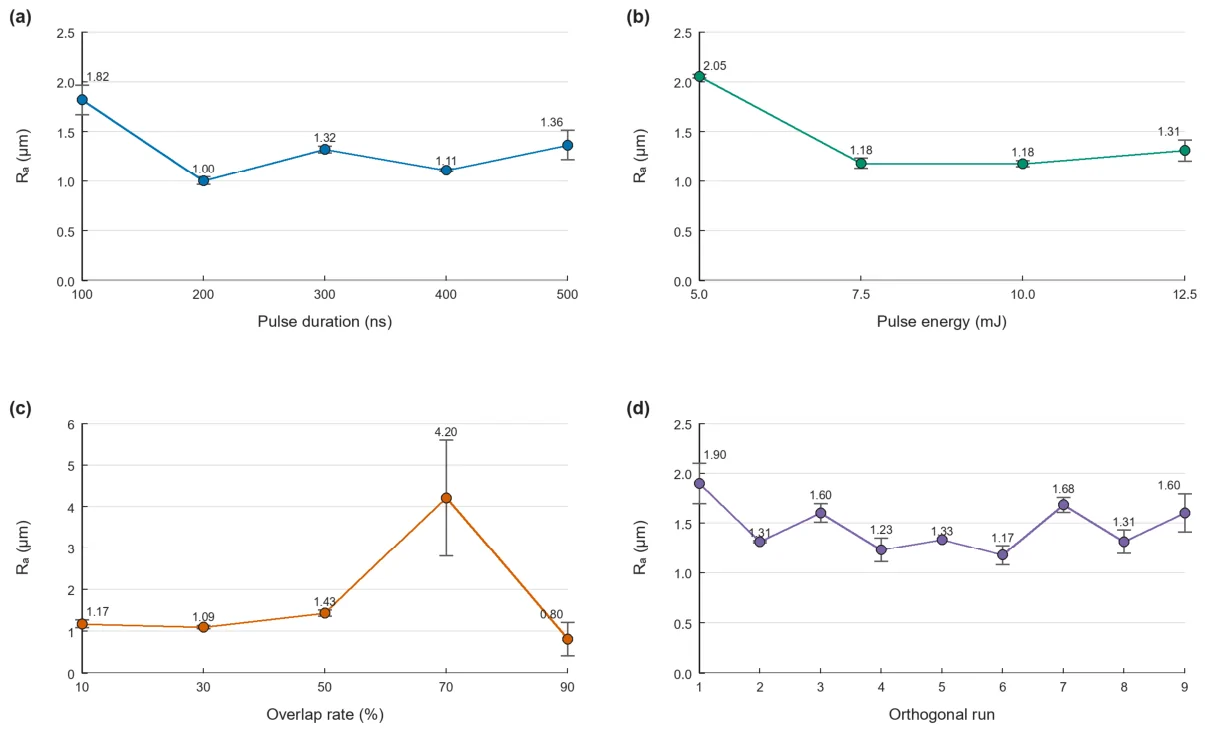
Surface roughness under single-factor and orthogonal laser-cleaning conditions: (**a**) pulse duration; (**b**) pulse energy; (**c**) overlap rate; and (**d**) L9 orthogonal runs. Data points are means of two to three valid traces on a representative coupon for each condition; error bars show the sample standard deviation among those traces.

Surface roughness was used as an additional surface-integrity index to complement PRR, SEM, and EDS. The averaged Ra values from valid within-surface traces are summarized in Figure 7, and the descriptive orthogonal range analysis is listed in Table 8. Error bars in Figure 7 represent trace-to-trace variation on the same coupon; they do not quantify between-coupon process repeatability. Ra was not forced into a monotonic trend because it can reflect residual coating, laser-generated pits, oxygen-bearing products, and local re-solidification.

In the pulse-duration series, Ra decreased from 1.816 μm at 100 ns to 1.001 μm at 200 ns and then fluctuated between 1.109 and 1.362 μm. This non-monotonic behavior shows that increasing pulse width does not simply smooth the surface. Short pulses can generate localized ablation pits, whereas intermediate pulse widths promote more continuous coating detachment. The slight Ra increase at 500 ns is consistent with greater thermal input and possible residual, carbonized, oxygen-bearing, or re-solidified material.

For the pulse-energy series, the lowest-energy condition gave the highest roughness (Ra = 2.053 μm), which is attributed to incomplete removal and an uneven carbonized/residual coating layer. Increasing the pulse energy to 7.5–10.0 mJ reduced Ra to approximately 1.18 μm, indicating a cleaner and more uniform removal state. A further increase to 12.5 mJ slightly increased Ra to 1.308 μm, suggesting that additional fluence mainly intensifies thermal loading rather than improving surface finish.

The overlap-rate series showed the strongest scatter. The 10% and 30% conditions had relatively low Ra values (1.170 and 1.094 μm), but PRR and EDS showed that these surfaces retained substantial coating. At 70% overlap, Ra increased to 4.196 μm, consistent with pronounced grooves, pits, or re-solidified features despite high PRR and Fe exposure. At 90% overlap, Ra decreased to 0.801 μm; this smoother profile should not be interpreted as better cleaning because SEM showed thermally affected and redeposited morphology. The low Ra may reflect smoothing by re-solidified or redeposited products rather than a cleaner metallic substrate.

The orthogonal experiment produced Ra values between 1.173 and 1.896 μm. Descriptive range analysis for Ra gives the apparent order pulse duration > power > overlap rate, which differs from the PRR order. The lowest predicted Ra combination is A2B2C2, whereas the tested Run 2, A1B2C2, gave Ra = 1.308 μm and PRR = 99.90%. A1B2C2 is therefore retained only as a measured engineering compromise among high removal efficiency, acceptable roughness, comparative residue indicators, and lower cumulative thermal input.

### 3.4. Surface Evolution and Cleaning Mechanism Revealed by SEM/EDS

#### 3.4.1. Evolution of Surface Morphology

**Figure 8:**
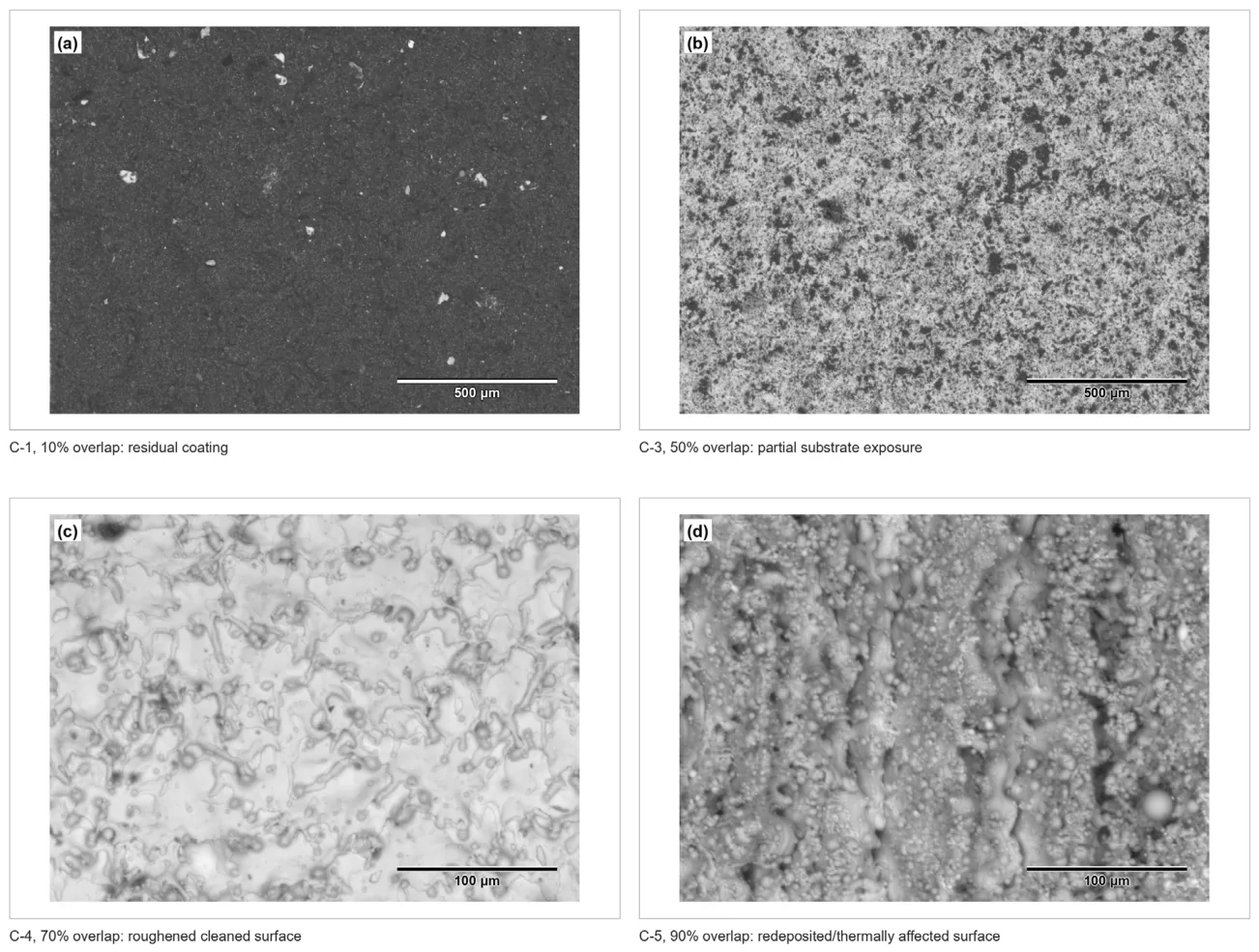
Representative SEM morphologies of AH36 steel after MOPA laser cleaning under different overlap conditions: (**a**) 10% overlap, residual coating; (**b**) 50% overlap, heterogeneous surface with partial substrate exposure; (**c**) 70% overlap, roughened surface; and (**d**) 90% overlap, re-solidified or redeposited features. Scale bars: 500 μm in (**a**,**b**) and 100 μm in (**c**,**d**).

Representative SEM observations compare four measured overlap conditions. Figure 8a shows the comparatively uniform residual layer at 10% overlap; Figure 8b shows a heterogeneous surface at 50% overlap with partial substrate exposure; Figure 8c shows pronounced roughened features at 70% overlap; and Figure 8d shows re-solidified or redeposited morphology at 90% overlap. SEM supports the distinction between residual coating, exposed substrate, and redeposited material, but it does not identify the oxide phase or chemical state.

At 10% overlap (Figure 8a), the high C/low Fe EDS result and the residual surface layer are consistent with laser action remaining largely within the coating. At 50% overlap (Figure 8b), the heterogeneous contrast and increased Fe signal indicate partial substrate exposure, while the source of the accompanying O signal remains unresolved because residual Fe_2_O_3_ pigment and thermally formed oxides cannot be separated by EDS.

At 70% overlap (Figure 8c), the surface is substantially exposed but visibly roughened, consistent with the high Ra value. This demonstrates why the high PRR and Fe exposure do not alone define a satisfactory surface. The morphology supports extensive coating removal, but it does not independently prove photothermal ablation, spallation, or oxide formation [21,23].

At 90% overlap (Figure 8d), re-solidified or redeposited features are prominent. Repeated irradiation plausibly increased heat accumulation and material redistribution. The morphology, lower PRR, and increased O signal jointly identify this condition as less favorable than moderate overlap, although the present SEM/EDS data cannot determine whether the oxygen belongs to residual Fe_2_O_3_ pigment, newly formed steel oxide, or oxygen-bearing redeposited products [4,5,25].

#### 3.4.2. Corrective Role of EDS in Evaluating Cleaning Quality

EDS composition did not always follow the same trend as macroscopic PRR. In the pulse-energy series, increasing fluence generally increased Fe and decreased C in the selected analysis areas, consistent with progressive removal of the organic matrix and greater substrate exposure. These comparative trends are useful for screening, but the single-area, semi-quantitative measurements do not provide a chemical-state identification or a statistical estimate of spatial variability.

In the overlap-rate series, PRR and EDS jointly show that C-1 and C-2 were insufficiently cleaned: PRR was 33.05% and 30.42%, Fe remained near 4.0 wt.%, and C/O signals were high. At 50% overlap, PRR rose to 97.72%, and C fell to 1.8 wt.%, while O was 43.4 wt.%. At 70% overlap, Fe reached 77.7 wt.%, and O decreased to 14.2 wt.%, but C increased to 8.1 wt.%, and Ra rose to 4.196 μm. At 90% overlap, the O signal increased to 32.3 wt.%, and SEM showed re-solidified or redeposited features. The latter condition is therefore less favorable than moderate overlap; however, the increased O cannot be assigned specifically to laser-induced oxidation because EDS cannot distinguish it from residual Fe_2_O_3_ pigment or oxygen-bearing redeposited material.

**Figure 9:**
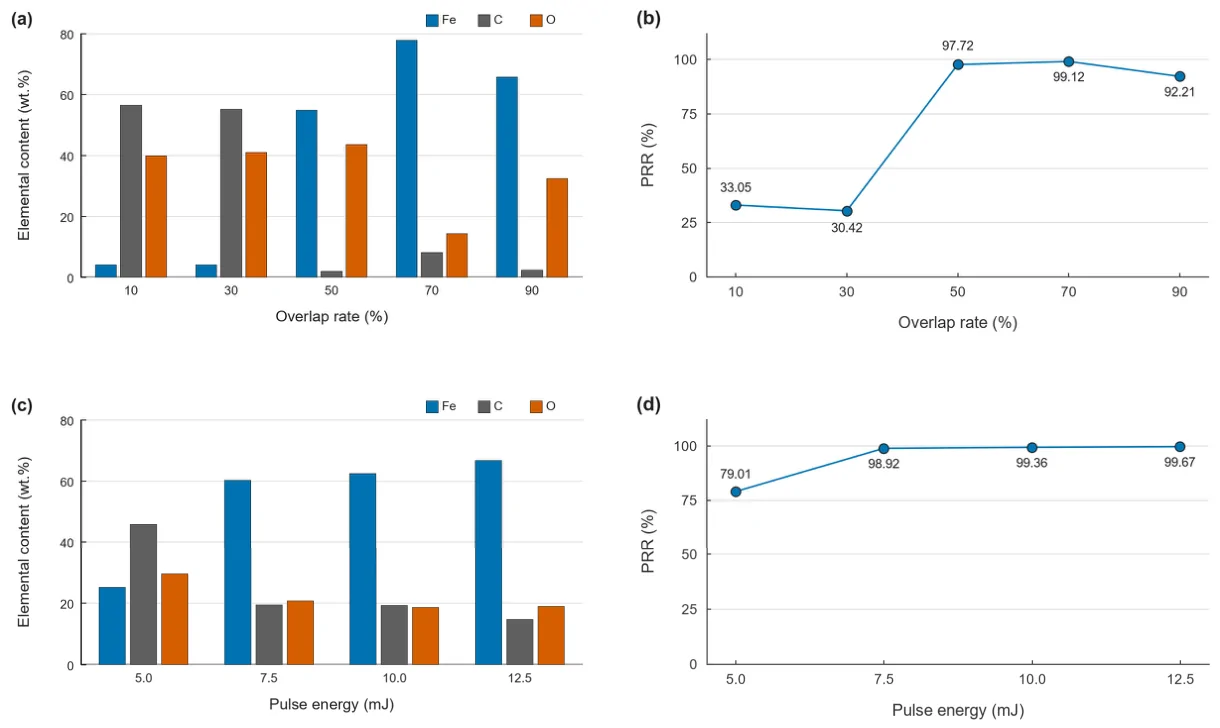
Coupled PRR and EDS evidence for process-window selection: (**a**) EDS composition versus overlap rate; (**b**) PRR versus overlap rate; (**c**) EDS composition versus single-pulse energy; and (**d**) PRR versus single-pulse energy.

Thus, EDS complements rather than replaces PRR and SEM. PRR describes image-based area change, EDS provides local elemental signals, and SEM reveals morphology. As summarized in Figure 9, a satisfactory condition requires agreement among high PRR, increased Fe exposure, reduced C, acceptable roughness, and morphology without pronounced residue or redeposition. O content is treated as a cautionary signal, not as proof of oxidation. Confirming the laser-induced oxide formation would require phase- or chemical-state-sensitive analysis such as XRD or XPS. Future work should also use multiple mapped areas and replicated coupons, followed by electrochemical or salt-spray testing after recoating [13,16,17].

#### 3.4.3. Mechanism of Laser Paint Removal

Based on the macroscopic morphology, PRR, EDS, and SEM evidence, the removal behavior of the 75 μm thick epoxy primer from AH36 steel can be explained by the combined contribution of surface photothermal ablation and interfacial thermo-mechanical spallation [2,7,21,22,23,24,27].

In the early stage of irradiation, the epoxy primer surface rapidly absorbs laser energy. The resin matrix undergoes thermal degradation, carbonization, and local gasification, producing high-temperature gases and ablation products. When the input energy or overlap is insufficient, the process remains mainly near the coating surface and cannot effectively reach the coating-steel interface. Consequently, only local ablation spots or carbonized residues form, and bulk removal is not achieved [22,23].

When energy density, pulse duration, and overlap are properly matched, heat transfer into the coating is likely to generate a strong transient temperature gradient near the coating-substrate interface. Because epoxy coatings and AH36 steel have markedly different thermal expansion coefficients, the resulting mismatch can produce interfacial thermal stress. When this stress becomes sufficient to overcome local coating adhesion, detachment as flakes or fragments may occur. This interpretation is consistent with efficient thick-coating removal while limiting excessive thermal effects on the exposed substrate [21,23].

When the laser input is increased, especially through high overlap, repeated exposure raises cumulative energy input and can roughen the surface and promote material redistribution. In the present data, the combination of increased O signal with pits, droplets, re-solidified ridges, or redeposited layers is used only as an empirical warning that the process may have moved outside the stable cleaning window. This conclusion does not depend on assigning the O signal to a specific oxide phase: residual pigment, thermally formed oxide, and oxygen-bearing redeposition can all indicate an undesirable surface state. Phase identification by XRD or XPS is required before claiming laser-induced oxidation as an established mechanism [4,5,24,25].

### 3.5. SEM Morphology of Representative Cleaning States

Representative SEM morphologies further illustrate the transition from incomplete coating removal to effective substrate exposure and, finally, to excessive thermal modification. At low energy input or insufficient overlap, residual coating remained on the AH36 surface, whereas moderate processing conditions produced greater substrate exposure with relatively limited surface damage. In contrast, excessive overlap or energy input resulted in pronounced surface roughening and re-solidified or redeposited features. These observations confirm that a high PRR alone is insufficient to define an appropriate cleaning condition, and that SEM morphology should be evaluated together with PRR, surface roughness, and EDS results when selecting the processing window.

### 3.6. Pull-Off Adhesion Performance After Recoating

**Figure 10:**
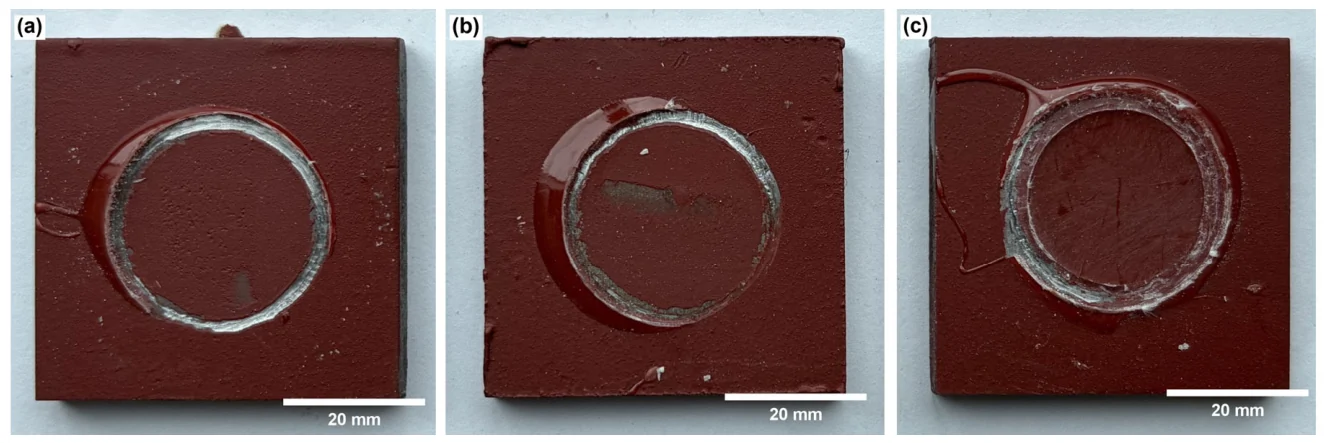
Representative pull-off test surfaces after recoating in accordance with ISO 4624:2016: (**a**) sandblasted reference, mean 5.91 MPa; (**b**) recommended laser-cleaned surface, mean 5.20 MPa; and (**c**) mechanically ground surface, mean 2.01 MPa (n = 3 dollies per condition). Coating remaining within the circular loading zones indicates predominantly cohesive failure within the epoxy coating. Scale bars: 20 mm.

Three independently positioned dollies were tested for each recoated surface condition (n = 3). The resulting mean pull-off strengths were 5.91 MPa for the sandblasted reference, 5.20 MPa for the selected laser-cleaned surface, and 2.01 MPa for the mechanically ground surface (Figure 10). Based on these mean values, the laser-cleaned condition retained 88.0% of the sandblasted reference strength and was 2.59 times the ground value. The individual dolly readings were not retained in the worksheet used for manuscript revision, so a standard deviation and formal statistical comparison cannot be reconstructed; the ratios are therefore descriptive and do not establish equivalence between surface preparations.

Visual examination of the detached areas showed that the red epoxy coating remained over most of the circular loading zones in all three specimens, without extensive clean exposure of the AH36 substrate. The fracture mode was therefore classified as predominantly cohesive failure within the coating rather than adhesive failure at the steel–coating interface. Under this failure mode, the recorded value represents the pull-off resistance of the recoated system and primarily reflects cohesive rupture within the coating; it is not a direct measurement of steel–coating interfacial adhesion. The steel–recoat interface was not the preferential failure location in any of the three specimens.

Accordingly, the three-dolly pull-off results provide preliminary evidence that the selected laser-cleaned surface is compatible with recoating. The cohesive fracture pattern suggests that the steel–recoat interface was not the preferential failure location in the tested dollies, but the available summary data do not quantify interfacial adhesion or demonstrate that laser cleaning is statistically equivalent to sandblasting. Future work should retain the individual dolly values, report mean ± standard deviation and failure-mode fractions, and use an appropriate statistical comparison. Service durability would additionally require corrosion testing after recoating [13,14,15].

## 4. Conclusions

In this study, a 1000 W MOPA pulsed fiber laser was used to remove a 75 μm thick iron-red epoxy primer from AH36 marine high-strength steel. Single-factor and orthogonal experiments were evaluated using PRR, Ra, EDS, SEM morphology, and recoating pull-off testing. The main conclusions are as follows:

(1) Pulse duration, power, and overlap rate jointly influenced coating removal. Each L9 combination was repeated on five independent coupons. Based on the retained run means, the apparent descriptive PRR order was overlap rate > power > pulse duration, with ranges of 6.77%, 6.30%, and 6.02%, respectively. Because replicate-level PRR values were not retained, these close range differences could not be tested against within-run error and should not be interpreted as a statistically established ranking.
(2) A1B2C2 (300 ns, 6.00 mJ at 100 kHz, and 60% overlap) was the already tested Run 2 and produced mean PRR = 99.90% and Ra = 1.308 μm. It was selected from the measured L9 combinations as a multi-index engineering compromise, not as an extrapolated or statistically validated optimum. Retaining complete replicate-level data in future confirmation tests is necessary before defining an industrial process window.
(3) Cleaning quality could not be determined from PRR or Fe exposure alone. At 70% overlap, Fe was 77.7 wt.%, C was 8.1 wt.%, and Ra was 4.196 μm; at 90% overlap, SEM showed re-solidified or redeposited features. The results are consistent with coupled photothermal removal and thermo-mechanical spallation, but EDS O signals do not prove laser-induced oxidation because residual Fe_2_O_3_ pigment and oxygen-bearing redeposition are alternative sources.
(4) Mean pull-off strengths based on three dollies per condition were 5.91 MPa for the sandblasted reference, 5.20 MPa for the selected laser-cleaned surface, and 2.01 MPa for the ground surface. Predominantly cohesive failure was observed. These results support recoating compatibility, but the absence of retained individual dolly values prevents calculation of standard deviations and formal statistical comparison.

## Figures and Tables

**Table 1:** Chemical composition of AH36 marine high-strength steel (wt.%).

Element	C	Mn	Si	P/S	Fe
Content (wt.%)	≤0.18	0.90–1.60	0.10–0.50	≤0.035	Balance

**Table 2:** Main chemical constituents of the iron-red epoxy coating.

Component	Chemical Type	Content (wt.%)	Primary Function	Remark
Epoxy Resin	Bisphenol-A (E-44)	30–40	Film-forming binder	Main matrix
Pigment	Iron oxide (Fe_2_O_3_)	25–35	Anti-corrosion	Rust inhibitor
Curing Agent	Polyamide	10–15	Cross-linking agent	Hardener
Fillers	Talc/BaSO_4_	15–25	Physical reinforcement	Extender
Additives	Various	1–5	Leveling and defoaming	Processing aid

**Table 3:** Key parameters of the MOPA pulsed fiber laser system.

Parameter	Value/Range	Unit
Laser type	MOPA pulsed fiber laser	—
Central wavelength	1060–1080	nm
Maximum average power	1000	W
Max single-pulse energy	15 (at τ = 500 ns)	mJ
Pulse duration (tunable)	100–500	ns
Repetition frequency	66–1000	kHz
Focused spot diameter	~250	μm
Galvanometer max speed	20,000	mm/s
Step length (hatch)	0.025–0.225	mm
F-theta focal length	160	mm
Output interface	QBH	—
Working distance	160	mm
Beam incidence angle	90 (normal incidence)	°

**Table 4:** Parameter gradients for single-factor experiments.

Group	Variable Parameter	Levels	Fixed Parameters	Note
A	Pulse duration τ (ns)	100, 200, 300, 400, 500	E = 6.25 mJ (power varies with f) η = 50%	f adjusted to maintain constant E per pulse; Vx matched to ηx = 50%
B	Single-pulse energy E (mJ)/Power (%)	5.0/40% 7.5/60% 10.0/80% 12.5/100%	τ = 400 ns; f = 80 kHz η = 50%	Ed ≈ 10.2, 15.3, 20.4, 25.5 J/cm^2^
C	Overlap rate η (=ηx = ηy)	10%, 30%, 50%, 70%, 90%	τ = 400 ns; f = 80 kHz Power = 60% (E = 7.5 mJ)	Vx and Ly adjusted proportionally to maintain isotropic η

**Table 5:** Orthogonal experimental array L9(3^3^): factors, levels, and response metrics.

Run	A: τ (ns)	B: Power (%)	C: η (%)	f (kHz)	E (mJ)	PRR (%)	Ra (μm)
1	300	50	50	100	5.00	80.86	1.896
2	300	60	60	100	6.00	99.90	1.308
3	300	70	70	100	7.00	99.15	1.601
4	400	50	60	80	6.25	99.95	1.227
5	400	60	70	80	7.50	98.65	1.331
6	400	70	50	80	8.75	98.32	1.173
7	500	50	70	66	7.58	98.67	1.682
8	500	60	50	66	9.09	99.84	1.309
9	500	70	60	66	10.61	99.47	1.600

**Table 6:** EDS elemental composition and paint removal rate of laser-cleaned surfaces at different overlap rates.

Specimen	Overlap Rate (%)	Fe (wt.% ± σ)	C (wt.% ± σ)	O (wt.% ± σ)	PRR (%)
C-1	10	4.0 ± 0.1	56.3 ± 0.7	39.7 ± 0.7	33.05
C-2	30	3.9 ± 0.1	55.1 ± 0.6	40.9 ± 0.6	30.42
C-3	50	54.7 ± 0.6	1.8 ± 0.8	43.4 ± 0.5	97.72
C-4	70	77.7 ± 0.7	8.1 ± 0.8	14.2 ± 0.4	99.12
C-5	90	65.5 ± 0.8	2.2 ± 1.0	32.3 ± 0.5	92.21

Note: σ is the EDS software-reported 1σ quantification uncertainty for one mapped field; it is not a between-coupon standard deviation.

**Table 7:** Range analysis of PRR in the L9 orthogonal experiment.

Factor	Level 1	k1 (%)	Level 2	k2 (%)	Level 3	k3 (%)	R (%)	Rank
A: Pulse duration	300 ns	93.30	400 ns	98.97	500 ns	99.33	6.02	3
B: Power	50%	93.16	60%	99.46	70%	98.98	6.30	2
C: Overlap rate	50%	93.01	60%	99.77	70%	98.82	6.77	1

**Table 8:** Range analysis of Ra in the L9 orthogonal experiment.

Factor	Level 1	k1 (μm)	Level 2	k2 (μm)	Level 3	k3 (μm)	R (μm)	Rank
A: Pulse duration	300 ns	1.602	400 ns	1.244	500 ns	1.530	0.358	1
B: Power	50%	1.602	60%	1.316	70%	1.458	0.286	2
C: Overlap rate	50%	1.459	60%	1.378	70%	1.538	0.160	3

## Data Availability

The original contributions presented in this study are included in the article. Further inquiries can be directed to the corresponding authors.

## References

[1] Li X.Y., Wang H.Y., Yu W.J., Wang L.Q., Wang D.W., Cheng H.X., Wang L.H. (2021). Laser paint stripping strategy in engineering application: A systematic review. Optik.

[2] Zhu G.D., Xu Z.H., Jin Y., Chen X., Yang L.J., Xu J., Shan D.B., Chen Y.B., Guo B. (2022). Mechanism and application of laser cleaning: A review. Opt. Lasers Eng..

[3] Shan T., Wang S.J., Yin F.S., Qiao Y.L., Liu P.F. (2021). A review of the application of laser cleaning and its influences on the substrate surface integrity. Mater. Rep..

[4] Hou L., Yin F., Wang S., Sun J., Yin H. (2024). A review of thermal effects and substrate damage control in laser cleaning. Opt. Laser Technol..

[5] Wang X., Yu J., Liu Y., Sun L., Li Y., He J., Mo Z., Wang B. (2021). Influence of spot overlapping in laser paint stripping with moving repetitive pulses. J. Laser Appl..

[6] Yang J., Zhou J., Sun Q., Meng X., Guo Z., Zhu M. (2021). Digital analysis and prediction of the topography after pulsed laser paint stripping. J. Manuf. Process..

[7] Zhao H., Zhang Q., Ma G., Qiao Y., Wang H. (2023). Numerical and experimental investigations for stripping mechanism of polyacrylate resin-based paint with a pulsed laser. Appl. Phys. A.

[8] Ma M.L., Wang L.M., Li J.F., Jia X.J., Wang X., Ren Y., Zhou Y.S. (2020). Investigation of the surface integrity of Q345 steel after Nd:YAG laser cleaning of oxidized mining parts. Coatings.

[9] Sonoiki D.S., Lapre C., Schnohr M., Scheibel K., Bang O., Petersen C.R. (2025). Comparing LIBS of marine coatings using femtosecond pulses and continuous wave laser cleaning. Spectrochim. Acta Part B At. Spectrosc..

[10] Li Z., Wang S., Zheng W., Wang Y., Pan Y. (2024). A review of dynamic monitoring technology and application research of laser cleaning interface. Measurement.

[11] Han H., Liu W.G., Zhang K. (2025). A study on the influence of laser parameters on the cleaning effect of metal coatings. J. Mater. Eng. Perform..

[12] Li Z., Wang S.J., Pan Y.K., Sun J.Z., Yin F.S. (2025). Construction of the heterogeneous coating model and multi-physics coupling analysis in laser cleaning. Opt. Laser Technol..

[13] Liu T., Ma L.W., Wang X., Wang J.K., Qian H.C., Zhang D.W., Li X.G. (2021). Self-healing corrosion protective coatings based on micro/nanocarriers: A review. Corros. Commun..

[14] Wang J.K., Wu S.H., Ma L.W., Zhao B., Xu H., Ding X.L., Zhang D.W. (2022). Corrosion resistant coating with passive protection and self-healing property based on Fe3O4-MBT nanoparticles. Corros. Commun..

[15] Ma Y.X., Jiang D., Yang Y.P., Ma L., Zhou J., Huang G.S., Lin C.G. (2023). Synthesis of magnetic targeted delivery microcapsules and its anti-corrosion self-healing behavior in epoxy resin coatings. Corros. Commun..

[16] Pan J.Q., Liu F.R., Feng J., Meng F.D., Chen Y.F., Chi J.N., Li Z.L., Li J., Liu L. (2025). Accurate recognition of micromorphology images of epoxy coatings for deep-sea environments based on a deep learning super-resolution method. Corros. Commun..

[17] Wang Y., Lu S.H., Xue N.T., Chen S.Q., Liu G.Z., Dou W.W. (2025). Deterioration mechanism of epoxy coating on EH36 ship steel during seawater freeze-thaw cycles. Corros. Commun..

[18] (2007). Preparation of Steel Substrates Before Application of Paints and Related Products—Visual Assessment of Surface cleanliness—Part 1: Rust Grades and Preparation Grades of Uncoated Steel Substrates and of Steel Substrates After Overall Removal of Previous Coatings.

[19] (2012). Preparation of Steel Substrates Before Application of Paints and Related Products—Surface Roughness Characteristics of Blast-Cleaned Steel Substrates—Part 2: Method for the Grading of Surface Profile of Abrasive Blast-Cleaned Steel—Comparator Procedure.

[20] (2016). Paints and Varnishes—Pull-Off Test for Adhesion.

[21] Lu Y., Yang L.J., Wang M.L., Wang Y. (2020). Improved thermal stress model and its application in ultraviolet nanosecond laser cleaning of paint. Appl. Opt..

[22] Zhang D., Xu J., Li Z., Jin Y., Su X., Shan D., Guo B. (2022). Removal mechanisms of nanosecond pulsed laser cleaning of blue and red polyurethane paint. Appl. Phys. A.

[23] Li Y., Li J., Zhang W., Jin G. (2024). Application of pulsed laser ablation thermal model in nanosecond pulsed laser removal of the epoxy resin paint film. Opt. Laser Technol..

[24] Lu Y., Ding Y., Wang M., Yang L., Wang Y. (2021). A characterization of laser cleaning painting layer from steel surface based on thermodynamic model. Int. J. Adv. Manuf. Technol..

[25] Zhu M., Zhang H., Shi K., Hou X., Shi Y., Fan D., Zhang W. (2024). Formation and effect of rough surface in pulsed laser removal of thick paint layer on 2024 aluminum. J. Laser Appl..

[26] Ma X., Zhang Y., Hou X., Ren Y., Xu Z., Cheng W., Qin X., Guo W., Zhang Q. (2025). Multi-objective optimization of laser cleaning quality of composite paint layers based on response surface. Coatings.

[27] Ji J.H., Shao G.P., He Y.Y., Tang F.Q., Fu H., Zhang Y.H. (2026). The removal mechanism of surface coatings from aluminum alloys via high-power nanosecond pulsed laser interlaced cleaning. Int. J. Adv. Manuf. Technol..

